# Sirt3 Regulates Response to Oxidative Stress by Interacting with BER Proteins in Colorectal Cancer

**DOI:** 10.1155/2022/7299555

**Published:** 2022-04-07

**Authors:** J. Kabzinski, A. Walczak, I. Majsterek

**Affiliations:** Department of Clinical Chemistry and Biochemistry, Medical University of Lodz, Lodz, Poland

## Abstract

The oxidative damages are well-recognized factors in the pathogenesis of colorectal cancer (CRC). Increased levels of reactive oxygen species (ROS) can lead to oxidative DNA damage, which, if unrepaired, can be an underlying cause of cancerogenic transformation. To defend against these threats, cells have developed a range of defense mechanisms. One of the most important protection mechanisms is DNA repair systems, both nuclear and mitochondrial. Sirt3 is a mitochondrial protein involved in regulating NEIL1, NEIL2, MUTYH, APE1, and LIG3 proteins, which are involved in DNA repair, including mitochondrial repair through mtBER (mitochondrial Base Excision Repair). In this work, we show that NEIL1, NEIL2, MUTYH, APE1, and LIG3 are regulated by Sirt3 through deacetylation, and moreover, Sirt3 is directly involved in physical interaction with MUTYH, NEIL1, and APE1, which indicates the controlling role of Sirt3 over the mtBER mechanism. Also, if the cells deprived of Sirt3 are exposed to oxidative stress, altered levels of those proteins can be observed, which supports the theory of the regulatory role of Sirt3. Finally, to fully confirm the role of Sirt3 in DNA repair, we examined its role in apoptosis and found the impact of this protein on cell survival rate. Using the knowledge obtained in the course of conducted experiments, we postulate consideration of Sirt3 as a target in the rising vulnerability of cancer cells during therapy and therefore increasing the effectiveness of cancer treatment.

## 1. Introduction

Despite improved diagnostic programs and increasingly advanced treatments, the incidence of colorectal cancer (CRC) continues to increase [1, 2]. In addition, direct causes of disease development still remain unknown, and the spectrum of factors contributing to the modulation of its risk is becoming increasingly widespread. As with other types of malignant tumors, the most important role seems to be the combination of genetic predispositions with the influence of environmental factors. Among the latter, the oxidative damages generated by oxidative stress are well-recognized factors. Reactive oxygen species (ROS) interacting with DNA lead to oxidative DNA damage, which eventually can result in the accumulation of mutations and as a consequence, a cancer transformation. To defend against these threats, cells have developed a range of defense mechanisms, such as antioxidant defense systems and DNA repair systems. Previously, it was believed that all these mechanisms work independently of each other. However, current reports indicate that some factors can participate in more than one mechanism at a time, and their role can be much more complex and complicated than previously thought.

Sirt3 is mitochondrial protein involved in regulating metabolic processes. Moreover, in addition to controlling metabolism at the transcriptional level, Sirt3 also directly controls the activity of metabolic enzymes [3–5]. Endonuclease VIII-like 1 (NEIL1), Endonuclease VIII-like 2 (NEIL2), mutY DNA glycosylase (MUTYH), Apurinic/apyrimidinic endonuclease 1 (APE1), and DNA ligase 3 (LIG3) are proteins involved in DNA repair, including mitochondrial repair through mtBER (mitochondrial Base Excision Repair) pathway; however, their role may go beyond this function and it is suspected that in addition to the repair function, they may play a role in response to stressors and be important components that maintain mitochondrial integrity [6, 7]. The role of the proteins selected for the experiment in DNA repair processes is NEIL1 and NEIL2—initiate the first step in base excision repair by cleaving bases damaged by reactive oxygen species and introducing a DNA strand break via the associated lyase reaction [8, 9] MUTYH glycosylase which excises adenine bases from the DNA backbone at sites where adenine is inappropriately paired with guanine, cytosine, or 8-oxo-7,8-dihydroguanine and [10], APE1—repair of damaged or mismatched nucleotides, major AP endonuclease in human cells [11] and Lig3, an ATP-dependent DNA ligase that seals interruptions in the phosphodiester backbone of duplex DNA [12].

The aim of this study was to examine the potential role of Sirt3 in the regulation of NEIL1, NEIL2, MUTYH, APE1, and LIG3 mitochondrial proteins by assessing their physical interactions, influence on activity, cooperation in oxidative damage removal, and finally role in apoptosis process in colorectal cancer (CRC) cells. Confirmation of the regulatory mechanism operated by Sirt3 within the mtBER will allow for a better understanding of the processes taking place within this pathway and its potential therapeutic use.

## 2. Materials and Methods

### 2.1. Cell Culture

Experiments were performed on the HT-29 human colon cancer cell line (ATCC). All the cell cultures were maintained in a 5% CO_2_ humidified incubator at 37°C in EMEM Medium (ATCC) with the addition of FBS (to a final concentration of 10% v/v) and penicillin and streptomycin (100 U/ml).

### 2.2. Transfection and Silencing/Overexpression of *Sirt3* Gene

Transfection was performed as described in our previous study [13]. Silencing Sirt3 gene: cells were treated with Sirt3 shRNA Lentiviral Particles Transduction (Santa Cruz) according to manufacturer's instruction. Overexpression of Sirt3 gene: cells were treated with pcDNA3.1-Flag-SIRT3 plasmid obtained from ADDGENE (Cambridge, MA, USA). Transfected cells, in addition to silencing *Sirt3*, acquire resistance to puromycin, which, through subsequent culturing in a medium with the addition of puromycin, allows only transfected cells to survive.

### 2.3. Deacetylation Assay

Control cells were transfected with Flag-Sirt3 plasmid and test cells with Flag-NEIL1, Flag-NEIL2, Flag-MUTYH, Flag-APE1, and Flag-LIG3 plasmids. Cells were then lysed in lysis buffer (50 mM Tris-HCl (pH 7.4), 150 mM NaCl, 1 mM EDTA, 1% Triton X-100) with the addition of a protease inhibitor at room temperature for 20 minutes and then centrifuged at room temperature. 12,000 xg for 20 minutes. Test proteins were purified using immunopurification (ANTI-FLAG M2 Gel, Sigma). In the deacetylation test, purified mtBER proteins were incubated with the Sirt3-Flag protein in the presence or absence of 1 mM NAD^+^ in deacetylase buffer (50 mM Tris-HCl (pH 9.0), 4 mM MgCl2, 50 mM NaCl, 0.5 mM dithiothreitol) at 30°C for 3 hours, and then the level of acetylated lysine residues in the tested proteins was measured using the ELISA test with Acetyl-Lysine Antibody LS-C71873 (LifeSpan Biosciences) that recognizes Acetylated-Lysine proteins only when posttranslationally modified by acetylation on the epsilon-amino groups of lysine residues. Acetylated lysine level was treated as a measure of deacetylation compared to control.

### 2.4. Inducing Oxidative Stress

Oxidative stress was induced by the addition of antimycin A (Sigma Aldrich) to the medium and subsequent incubation for 1, 3, 24, and 48 hours. Final concentrations of oxidative stress factor: 10 *μ*M and 100 *μ*M.

### 2.5. Protein Isolation

Total protein was obtained by cells lysis in EBC buffer (50 mM Tris pH 8.0; 120 mM NaCl; 0.5% NP-40) supplemented with protease and phosphatase inhibitors.

Mitochondrial protein was obtained by isolating mitochondrion from cultured cells with Mitochondria Isolation Kit for Cultured Cells (Thermo Scientific), followed by protein isolation.

### 2.6. Western Blot Assay

Cells were lysed in EBC buffer (50 mM Tris pH 8.0; 120 mM NaCl; 0.5% NP-40) supplemented with protease and phosphatase inhibitors. Total protein (100 *μ*g) was resolved on denaturing 10% polyacrylamide gels, transferred to nitrocellulose membranes or PVDF (Millipore), and blotted with the indicated primary antibodies: Sirt3, NEIL1, NEIL2, MUTYH, APE1, and LIG3 (Cell Signaling). Sites of antibody binding were visualized by an enhanced chemiluminescence detection kit (Bio-Rad). Protein levels were measured after Western Blotting by optical density readings with Quantity One 4.6.3 (Bio-Rad) software. Beta actin was used for normalization.

### 2.7. Terminal Deoxynucleotidyl Transferase dUTP Nick End Labeling (TUNEL)

Detection of apoptosis was done with the usage of the TiterTACS kit (R&D Systems) according to manufacturer's protocol, and results were read using a microplate reader (Synergy). In order to induce DNA damage, cells were treated with gamma radiation at a dosage of 25 Gy.

### 2.8. Statistical Analysis

To compare protein levels between control, silenced Sirt3 and Sirt3 overexpression groups, a single factor one-way ANOVA test (analysis of variance) was performed. If the test revealed that the means of the three groups were not all equal, a *t*-Test to test each pair of means was performed. At first, an F-test to determine if the variances of the two populations are equal was done, and depending on the result, a Two-Sample Assuming Unequal Variances *t*-Test or Two-Sample Assuming Equal Variances *t*-Test was performed.

## 3. Results

### 3.1. Role of Sirt3 in NEIL1, NEIL2, MUTYH, APE1, and LIG3 Deacetylation

**Figure 1:**
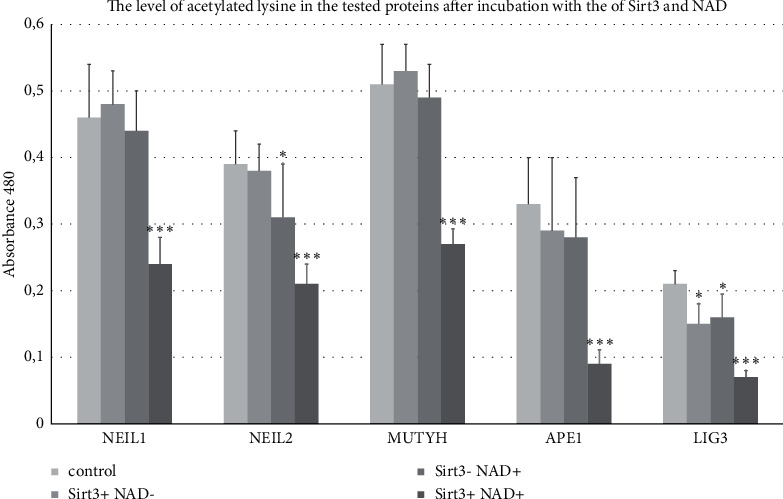
Acetylated lysine level in tested proteins after incubation with a mixture of Sirt3 and NAD (^*∗∗∗*^—*p* < 0.01; ^*∗*^—*p* < 0.05).

As a result of testing the ability of Sirt3 to deacetylate the studied proteins, it was found that for NEIL1, NEIL2, MUTYH, APE1, and LIG3, the level of acetylated lysine after incubation with Sirt3 and its NAD^+^ cofactor was significantly lower than in control. In addition, in order to confirm the need for the presence of both Sirt3 showing enzymatic activity of deacetylase and its NAD^+^ cofactor, incubations of the tested proteins were performed in the option: only Sirt3, only NAD^+,^ and Sirt3 + NAD^+^. A significant decrease in acetylated lysine was observed when incubating NEIL1, NEIL2, MUTYH, APE1, and LIG3 with both Sirt3 and NAD^+^. Because lysine is an excellent target for acetylases, its amount in the reaction mixture is a derivative of Sirt3 activity as deacetylase. The reduced level of acetylated lysine indicates that the tested proteins may be a substrate for Sirt3 in the presence of NAD^+^. Levels of acetylated lysine in the tested proteins after incubation with Sirt3 and NAD^+^ are shown in Figure 1.

### 3.2. Physical Protein Interaction Between Sirt3 and NEIL1, NEIL2, MUTYH, APE1, and LIG3 Proteins

**Figure 2:**
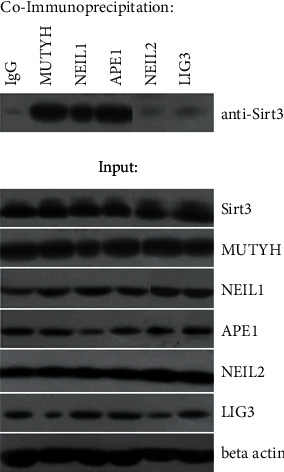
Coimmunoprecipitation of Sirt3 with MUTYH, NEIL1, and APE1 proteins and lack of coimmunoprecipitation with NEIL2 and LIG3. Coimmunoprecipitation section shows the presence or absence of physical interaction after incubation with antibodies against the indicated protein (with IgG as negative control) followed by incubation with anti-Sirt3. Input section shows the presence of protein after treatment with indicated antibody without coimmunoprecipitation.

To further explore the interaction between Sirt3 and tested proteins, a series of coimmunoprecipitation experiments were performed. In these experiments, cell lysates were immunoprecipitated by IgG or anti- NEIL1, NEIL2, MUTYH, APE1, and LIG3 protein and then immunoblotted with anti-Sirt3 antibody to check for physical protein interaction. Sirt3 was coimmunoprecipitated in samples incubated with the anti-MUTYH, NEIL1, and APE1 antibodies, indicating the interaction between Sirt3 and these three proteins. The image showing coimmunoprecipitation of Sirt3 and NEIL1, MUTYH, and APE1 is shown in Figure 2. No coimmunoprecipitation was observed for NEIL2 and LIG3.

### 3.3. Levels of NEIL1, NEIL2, MUTYH, APE1, and LIG3 in Oxidative Stress in Case of Absence or Excess of Sirt3

**Figure 3:**
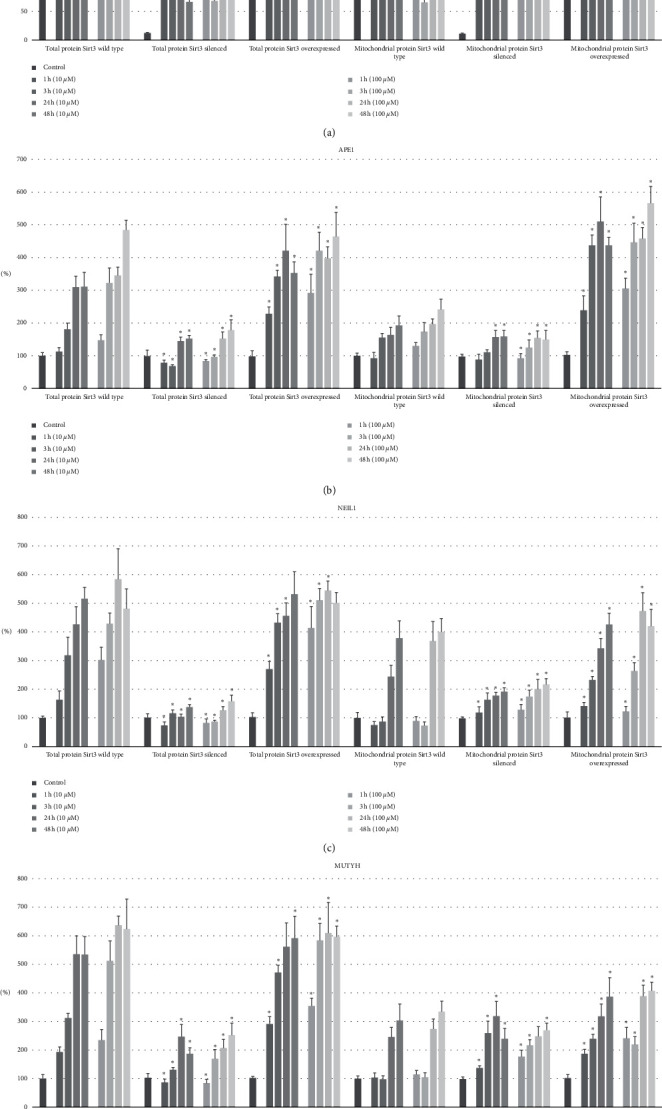
The effect of silencing/overexpressing the *Sirt3* gene on the level of BER proteins in total and mitochondrial protein after induction of oxidative stress with antimycin A (at final concentrations of 10 *μ*M and 100 *μ*M). The control for the *Sirt3* silenced and overexpression groups are indicative of the protein level with respect to the wild type. The percentage level is the change in the protein level after a given amount of time with respect to the control. ^*∗*^ The statistically significant difference (*p* < 0.05) between the increase/decrease in protein level with respect to the wild-type.

Further experiments aimed to assess the level of mtBER proteins in the absence or excess of Sirt3. This situation was achieved by silencing or overexpressing the Sirt3 gene in cell culture. In addition, oxidative stress conditions were used to assess the effectiveness of mtBER through the presence of repair proteins, which was induced by the addition of antimycin A to the medium followed by incubation for 1, 3, 24, and 48 hours. The final concentrations of oxidative stress factors were 10 *μ*M and 100 *μ*M. The studies were conducted on both total proteins isolated from cell lysate and on mitochondrial protein (protein isolation preceded by isolation of mitochondria from cell culture). Statistically significant differences were observed for the following proteins: APE1, NEIL1, and MUTYH and for those proteins, results are shown in Table 1 and visualized in Figure 3. First, the synthesis of new Sirt3 was assessed as a cell response to oxidative stress in the case of excess and deficiency of Sirt3 already present in the cell. For total protein, there was a statistically less significant increase in newly synthesized Sirt3 for both *Sirt3* silenced and Sirt3 overexpressed cultures as compared to the wild type (except at 10 *μ*M after 1 hour, where there was a greater increase). The opposite was true for the mitochondrial protein, where the rises were greater than in the wild type. Then, measurements of the mtBER proteins level were performed in order to evaluate the influence of the Sirt3 on their quantity and synthesis rate under oxidative stress conditions. For APE1, silencing *Sirt3* resulted in statistically significant (except for 1 hour and 24 hours for the 10 *μ*M concentration) lower protein gains for both total and mitochondrial protein. This effect was much more evident for total protein and was especially noticeable after the longest incubation time. For *Sirt3* overexpression, a hike in the amount of APE1 was observed compared to the wild type. However, in this case it occurred to a much greater extent for the mitochondrial protein. A similar trend occurred for NEIL1, where increases in total protein amount were significantly lower for silenced *Sirt3*. However, for the mitochondrial protein, decreases were observed only for longer times of exposure to oxidative stress (24 and 48 hours), while they were preceded by an increase in NEIL1 for shorter times (1 and 3 hours). For *Sirt3* overexpressing cultures, NEIL1 levels were significantly (except for 48 hours for both 10 and 100 *μ*M concentrations) higher for both total and mitochondrial protein, except for the 24 hours at 100 *μ*M where a reduction was observed. Finally, for MUTYH, the trend was similar. Total protein with *Sirt3* silenced had significantly lower levels than wild type. In contrast, the mitochondrial protein, similar to NEIL1, after initial elevations (for 1, 3, and 24 hours at 10 *μ*M and 1 and 3 hours for 100 *μ*M) was significantly lesser compared to wild type. For *Sirt3* overexpression, the level of MUTYH in total protein was notably (except 24 hours for 10 *μ*M) higher, but at 100 *μ*M only in the initial phases of incubation with the stress factor because after 24 and 48 hours, the level of MUTYH had increased less than in the wild-type. For MUTYH in the mitochondrial protein, no such distinction was observed. The levels were higher at each time point and concentration.

### 3.4. Influence of Sirt3 on Cell Survival

**Figure 4:**
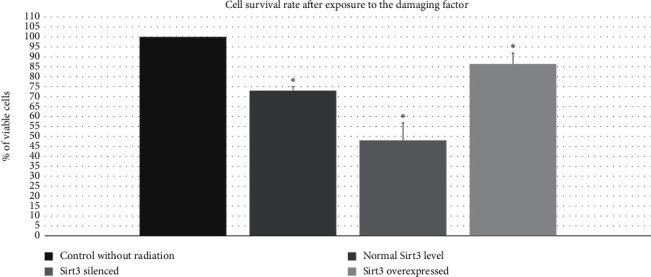
The level of apoptosis in cells exposed to the damaging factor (ionizing radiation at a dose of 25 Gy) in case of silencing/overexpressing of the Sirt3 gene (^*∗*^—*p* < 0.05).

After indicating the effect of Sirt3 on selected mtBER repair system proteins, the next step was to assess the effect of Sirt3 on the overall DNA repair system and thus its influence on modulation of cell survival. Terminal deoxynucleotidyl transferase dUTP Nick end labeling (TUNEL) test was used to assess the level of apoptosis after radiation-induced cell damage. 25 Gy dosage was used as suggested to us a few years ago by our colleagues - clinicians (from the colorectal surgery department) as the best reflecting the reality of the therapy. In the course of the experiment, three cell lines were established: in the first one, Sirt3 was silenced, in the second, the expression of Sirt3 was at a normal level and finally, in the third one, Sirt3 was overexpressed. After damage induction, the level of apoptosis was measured by the TUNEL method. For normal Sirt3 expression, a decrease in cell survival was observed. For cells with altered levels of Sirt3, when compared to the wild type, the degree of apoptosis was significantly higher in the case of silenced *Sirt3*, while for *Sirt3* overexpression, a significantly lower apoptosis rate was observed. Results are shown in Figure 4.

## 4. Discussion

Sirt3 is a mitochondrial protein with a wide spectrum of functions. Its primary role is to maintain mitochondrial integrity through enzyme regulation, a task achieved by deacetylation and acetylation of mitochondrial enzymes. Dysfunction of this mechanism is a recognized factor leading to the development of cancerous cells or apoptosis [14–16]. Therefore, Sirt3 is indirectly involved in protecting DNA from oxidative damage [17, 18] by the coordination of enzymatic activity and at the same time, directly participates in DNA repair through interaction with repair proteins, e.g., OGG1 [19]. All these aspects make Sirt3 a key protein in maintaining cellular stability, and its deficiency or aberrancy lies in the basis of many diseases, including cancers [20], especially since Sirt3 itself may act as a tumor-suppressor [21]. The Sirt3 control over DNA repair is very complex and includes both activation and inactivation of enzymes through deacetylation (as we have already indicated in our earlier study [13]) and direct physical interaction between proteins. In order to understand how broad the spectrum of action of Sirt3 is, we selected a set of DNA repair proteins and tested the interaction between Sirt3 and these proteins, the effect of *Sirt3* silencing and overexpression on the activity and efficiency of these proteins, as well as on the overall state of the cell after exposure to damaging factors. In the course of the research, we confirmed that all tested proteins can be a substrate for the deacetylation activity of Sirt3, which can thus regulate the mtBER process. These results are consistent with the available data that indicate the broad regulatory role of Sirt3 both in the regulation of mitochondrial enzymes through deacetylation [22] and the regulation of oxidative processes, whether associated with diseases of aging [23] or even oxidative lipid metabolism [24]. Available studies, however, indicate that deacetylation is not the only way Sirt3 regulates other proteins. It has been shown that such regulation can also take place through physical interaction between protein between Sirt3 and OGG1 [16]. Therefore, we checked whether this phenomenon occurs in the case of the selected mtBER proteins using the coimmunoprecipitation method. Direct physical interaction was found to occur between Sirt3 and MUTYH, NEIL1, and APE1. This sheds new light on the role of Sirt3 in repairing DNA damage and clearly indicates that this protein not only participates in the BER mechanism by activating the proteins in this process, but Sirt3 is also directly involved in removing the damage. Reports to date indicate that Sirt3 may interact directly with proteins such as an element of electron transport chain NADH dehydrogenase 1 alpha subcomplex subunit 9 [5] or FOXO3a O subclass protein of the forkhead family of transcription factors [25]. However, apart from the previously mentioned OGG1, such relationships were not observed for mtBER proteins before. Because the main activity of MUTYH, NEIL1, and APE1 is based on the removal of oxidative damage, these proteins can be considered as postfactum antioxidant mechanisms. Importantly, however, recent reports indicate a much wider role of these proteins than just participation in DNA repair. Especially in the case of APE1, it is suggested that it may be a factor involved in Redox Signaling [26] and has transcriptional regulatory activities [27]. Considering that these proteins should be classified not only as postfactum antioxidant systems but also as a mechanism actively involved in the antioxidant defense, we checked the effect of deficiency or excess of Sirt3 on their ability to remove DNA oxidative damage. In the cultures with silenced *Sirt3*, for APE1, NEIL1, and MUTYH, a significant decrease in the number of tested proteins was observed, especially for total protein. This clearly indicates the involvement of Sirt3 in the mtBER pathway, and we postulate that Sirt3 plays a role in activating the mechanism that removes oxidative damage, thus supplementing the previous reports on the role of Sirt3 in protecting mitochondrial DNA [11, 28]. At the same time, it should be noted that there must be an alternative way to activate the mechanism or that Sirt3 is not absolutely necessary for this activation since the mechanism works in the absence of Sirt3. However, its activation is slower and less efficient. This effect seems to be confirmed by the second version of the experiment in which *Sirt3* overexpression was used, in which case the number of tested proteins reached levels exceeding quantities observed for nontransfected cells, especially in the case of mitochondria. Available data suggest that Sirt3 cooperates with DNA repair systems to remove oxidative damage [29–31], and our results supplement it with recognition of Sirt3 direct effect on DNA repair proteins. And since high ROS levels restrict cancer cell survival [32], suppression of antioxidant systems could contribute to increasing cancer cells apoptosis. Presented interactions between Sirt3 and MUTYH, NEIL1, and APE1 in cancer cells and the effect of Sirt3 on the cellular response to oxidative stress indicate the necessity of Sirt3 for proper cell antioxidative reaction. We suggest that Sirt3 plays a role not only in the regulation of antioxidant mechanisms dealing with the threat directly but also prophylactically activates mechanisms that are designed to remove the effects of ROS action. This mechanism occurs throughout the cell and is replicated in the case of the mitochondrion. However, an exceptional situation occurs in the mitochondria in the case of NEIL1, whose level in the absence of Sirt3 initially rises, and only in the later stages of exposure to oxidative stress does it turn out to be lower than in wild-type cells. This situation is probably caused by the fact that NEIL1 has many different routes of activation [33, 34] and is a starter enzyme in the case of mitochondrial BER and therefore responds to oxidative damage as soon as possible. Sirt3's control over such a broad spectrum of proteins is reflected in apoptosis levels. When gamma radiation was used as the damaging factor, higher rates of apoptosis were observed with silenced *Sirt3*, whereas overexpression resulted in a lower level. However, the situation is very complicated, and literature reports indicate that Sirt3 can be both a proapoptotic factor [35, 36] and inhibit apoptosis [37]. It all depends on the state in which the cell is currently in, external factors, and, above all, the type of the cell. It should be emphasized that all our experiments were performed on colon cancer cell lines. Therefore, the results described in this article should be viewed through the prism of mechanisms occurring in CRC. Using the knowledge obtained in the course of conducted experiments, we postulate Sirt3 as a target in making cancer cells more vulnerable and increasing the effectiveness of cancer therapy, leading to a higher level of apoptosis. Such studies are conducted in the case of head and neck cancers [38] or lung cancers [39]. Available data suggest that Sirt3 is involved in cancer processes in CRC [40, 41], as in most other cancers, but without further detailed research, we still do not know whether Sirt3 is a tumor promoter or tumor suppressor in colorectal cancer [42].

## 5. Conclusions

NEIL1, NEIL2, MUTYH, APE1, and LIG3 are regulated by Sirt3 through deacetylation, and moreover, Sirt3 is directly involved in physical interaction with MUTYH, NEIL1, and APE1. Also, in case of those proteins, we observed altered levels after inducing oxidative stress in the case of silenced *Sirt3* gene. Finally, Sirt3 influences cell survival rate.

## Figures and Tables

**Table 1:** Effect of *Sirt3* gene silencing and overexpression on the levels of Sirt3, APE1, NEIL1, and MUTYH proteins in conditions of oxidative stress induced by antimycin A (at final concentrations of 10 *μ*M and 100 *μ*M). The control for the *Sirt3* silenced and overexpression groups are indicative of the protein level with respect to the wild type. The percentage level is the change in the protein level after a given amount of time with respect to the control. The statistically significant difference (*p* < 0.05) between the increase/decrease in protein level with respect to the wild-type is shown in bold.

Sirt3			10 *μ*M				100 *μ*M			
			Incubation time (hours)				Incubation time (hours)			
		Control	1	3	24	48	1	3	24	48
Sirt3 wild type	Total protein	1,000	0,854	1,453	2,035	2,036	1,235	1,935	1,834	2,135
		100%	85%	145%	203%	204%	124%	193%	183%	213%
	Mitochondrial protein	1,000	0,977	0,830	0,882	0,957	0,924	0,661	0,703	0,933
		100%	98%	83%	88%	96%	92%	66%	70%	93%
Sirt3 silenced	Total protein	0,123	**0,154**	**0,093**	**0,123**	**0,082**	**0,125**	**0,084**	**0,125**	**0,093**
		12%	**125%**	**76%**	**100%**	**67%**	**102%**	**69%**	**101%**	**76%**
	Mitochondrial protein	0,111	**0,154**	0,094	**0,152**	**0,135**	**0,124**	**0,098**	**0,095**	**0,152**
		11%	**139%**	85%	**137%**	**121%**	**112%**	**88%**	**86%**	**137%**
Sirt3 overexpressed	Total protein	3,347	**3,457**	**4,123**	**4,557**	**4,214**	**3,944**	**4,255**	**4,856**	**3,745**
		335%	**103%**	**123%**	**136%**	**126%**	**118%**	**127%**	**145%**	**112%**
	Mitochondrial protein	3,487	**4,346**	**4,463**	**5,395**	**6,582**	**4,568**	**5,136**	**6,236**	**6,782**
		349%	**125%**	**128%**	**155%**	**189%**	**131%**	**147%**	**179%**	**195%**

*APE1*			*10 μM*				*100 μM*			
			Incubation time (hours)				Incubation time (hours)			

		Control	1	3	24	48	1	3	24	48
Sirt3 wild type	Total protein	1,000	1,128	1,806	3,094	3,112	1,472	3,226	3,452	4,843
		100%	113%	181%	309%	311%	147%	323%	345%	484%
	Mitochondrial protein	1,000	0,923	1,555	1,635	1,926	1,298	1,734	1,966	2,413
		100%	92%	155%	163%	193%	130%	173%	197%	241%
Sirt3 silenced	Total protein	0,989	**0,777**	**0,673**	**1,428**	**1,508**	**0,829**	**0,951**	**1,507**	**1,765**
		98,9%	**79%**	**68%**	**144%**	**152%**	**84%**	**96%**	**152%**	**178%**
	Mitochondrial protein	0,973	**0,857**	**1,076**	1,529	**1,546**	**0,896**	**1,215**	**1,503**	**1,451**
		97,3%	**88%**	**111%**	157%	**159%**	**92%**	**125%**	**155%**	**149%**
Sirt3 overexpressed	Total protein	0,979	**2,235**	**3,346**	**4,123**	**3,456**	**2,856**	**4,123**	**3,890**	**4,546**
		97,9%	**228%**	**342%**	**421%**	**353%**	**292%**	**421%**	**397%**	**464%**
	Mitochondrial protein	1,023	**2,445**	**4,472**	**5,214**	**4,475**	**3,123**	**4,568**	**4,685**	**5,789**
		102,3%	**239%**	**437%**	**510%**	**437%**	**305%**	**447%**	**458%**	**566%**

*NEIL1*		*10 μM*				*100 μM*				
		Incubation time (hours)				Incubation time (hours)				

	Control	1	3	24	48	1	3	24	48	
Sirt3 wild type	Total protein	1,000	1,630	3,185	4,266	5,164	3,023	4,291	5,840	4,810
		100%	163%	319%	427%	516%	302%	429%	584%	481%
	Mitochondrial protein	1,000	0,746	0,868	2,439	3,783	0,895	0,728	3,682	3,997
		100%	75%	87%	244%	378%	90%	73%	368%	400%
Sirt3 silenced	Total protein	1,011	**0,744**	**1,173**	**1,048**	**1,388**	**0,835**	**0,878**	**1,277**	**1,599**
		101,1%	**74%**	**116%**	**104%**	**137%**	**83%**	**87%**	**126%**	**158%**
	Mitochondrial protein	0,981	**1,161**	**1,602**	**1,744**	**1,880**	**1,258**	**1,712**	**1,971**	**2,124**
		98,10%	**118%**	**163%**	**178%**	**192%**	**128%**	**174%**	**201%**	**216%**
Sirt3 overexpressed	Total protein	1,024	**2,765**	**4,429**	**4,672**	5,448	**4,236**	**5,234**	**5,577**	5,134
		102,4%	**270%**	**433%**	**456%**	532%	**414%**	**511%**	**545%**	501%
	Mitochondrial protein	1,008	**1,424**	**2,343**	**3,458**	**4,294**	**1,235**	**2,658**	**4,768**	**4,238**
		100,8%	**141%**	**232%**	**343%**	**426%**	**122%**	**264%**	**473%**	**420%**

*MUTYH*		*10 μM*				*100 μM*				
		Incubation time (hours)				Incubation time (hours)				

	Control	1	3	24	48	1	3	24	48	
Sirt3 wild type	Total protein	1,000	1,934	3,124	5,356	5,346	2,345	5,123	6,367	6,235
		100%	193%	312%	536%	535%	235%	512%	637%	623%
	Mitochondrial protein	1,000	1,034	0,973	2,457	3,035	1,143	1,043	2,736	3,344
		100%	103%	97%	246%	303%	114%	104%	274%	334%
Sirt3 silenced	Total protein	1,031	**0,893**	**1,346**	**2,545**	**1,923**	**0,864**	**1,745**	**2,134**	**2,598**
		103,1%	**87%**	**131%**	**247%**	**187%**	**84%**	**169%**	**207%**	**252%**
	Mitochondrial protein	0,981	**1,346**	**2,542**	**3,125**	**2,346**	**1,735**	**2,124**	2,434	**2,643**
		98,1%	**137%**	**259%**	**319%**	**239%**	**177%**	**217%**	248%	**269%**
Sirt3 overexpressed	Total protein	1,023	**2,976**	**4,824**	5,748	**6,046**	**3,624**	**5,975**	**6,235**	**6,105**
		102,3%	**291%**	**472%**	562%	**591%**	**354%**	**584%**	**609%**	**597%**
	Mitochondrial protein	1,018	**1,903**	**2,437**	**3,236**	**3,935**	**2,454**	**2,235**	**3,955**	**4,144**
		101,8%	**187%**	**239%**	**318%**	**386%**	**241%**	**219%**	**388%**	**407%**

## Data Availability

The data are available upon request from the corresponding author.
